# Metastasis is altered through multiple processes regulated by the E2F1 transcription factor

**DOI:** 10.1038/s41598-021-88924-y

**Published:** 2021-05-04

**Authors:** Matthew R. Swiatnicki, Eran R. Andrechek

**Affiliations:** 1 Department of Microbiology and Molecular Genetics, Michigan State University, East Lansing, MI 48824 USA; 2 Department of Physiology, Michigan State University, 2194 BPS Building, 567 Wilson Road, East Lansing, MI 48824 USA

**Keywords:** Cancer genomics, Cancer models, Metastasis

## Abstract

The E2F family of transcription factors is important for many cellular processes, from their canonical role in cell cycle regulation to other roles in angiogenesis and metastasis. Alteration of the Rb/E2F pathway occurs in various forms of cancer, including breast cancer. E2F1 ablation has been shown to decrease metastasis in MMTV-Neu and MMTV-PyMT transgenic mouse models of breast cancer. Here we take a bioinformatic approach to determine the E2F1 regulated genomic alterations involved in the metastatic cascade, in both Neu and PyMT models. Through gene expression analysis, we reveal few transcriptome changes in non-metastatic E2F1^−/−^ tumors relative to transgenic tumor controls. However investigation of these models through whole genome sequencing found numerous differences between the models, including differences in the proposed tumor etiology between E2F1^−/−^ and E2F1^+/+^ tumors induced by Neu or PyMT. For example, loss of E2F1 within the Neu model led to an increased contribution of the inefficient double stranded break repair signature to the proposed etiology of the tumors. While the SNV mutation burden was higher in PyMT mouse tumors than Neu mouse tumors, there was no statistically significant differences between E2F WT and E2F1 KO mice. Investigating mutated genes through gene set analysis also found a significant number of genes mutated in the cell adhesion pathway in E2F1^−/−^ tumors, indicating this may be a route for disruption of metastasis in E2F1^−/−^ tumors. Overall, these findings illustrate the complicated nature of uncovering drivers of the metastatic process.

## Introduction

Breast cancer is the most diagnosed cancer in women. To study genomic events contributing to breast cancer, numerous genetically engineered mouse models have been generated, including MMTV-Neu^1^ which recapitulates HER2+ve breast cancer, and MMTV-Polyoma virus Middle T antigen (PyMT)^2^. The PyMT model is highly aggressive, with tumors appearing at 45 days of age. Metastasis to the lung occurs in over 90% of tumor bearing mice, resulting in wide use of PyMT for metastasis studies. Similar to human breast cancers, both Neu and PyMT models have striking heterogeneity at histological and gene expression levels^3–6^, reinforcing the importance of these models as tools for the study of breast cancer.

Previous studies using Neu and PyMT models predicted a key role for the E2F1 transcription factor through a pathway signature analysis, suggesting that mechanisms outside the overexpression of the Neu or PyMT oncogene were contributing to tumor biology^7,8^. The E2F family of transcription factors is involved in numerous cellular processes, best known for cell cycle control. Usually sequestered by retinoblastoma (Rb), E2F1 is released to act on downstream targets upon Rb phosphorylation^9^. While mutations in E2F1 are not common in human breast cancer, mutations within the E2F pathway occur in over 25% of breast cancer patients, illustrating the importance of the pathway^10–14^.

To test the hypothesis that E2F1 regulates key events in Neu and PyMT tumors, E2F1 knockout (KO) mice^13^ were interbred with Neu and PyMT models^7,8^. This resulted in mammary tumors with changes in latency, growth rate, and a significant decrease in metastasis to the lung. Metastasis is the ultimate cause of mortality in cancer, with an estimated 90% of cancer deaths resulting from the spread of cancer cells to distal sites within the body^15^. Typically, cancer cells undergo numerous important steps for completion of the metastatic cascade. These include escape from the primary tumor, intravasation, extravasation, and seeding the distal site^16^ as reviewed by Welch^17^.

An important component contributing to the metastatic capability of a tumor is its microenvironment. Various collagens and proteins integral to cellular and tissue structure are capable of impacting metastatic potential. Indeed, proteins within the extracellular matrix, including collagen IV, have been found to regulate metastasis within the liver^18^. Collagen IV is a major component of the basement membrane, an important barrier to tumor invasion and breaching this has been shown to be a critical early step in tumor invasion and metastasis^19,20^. Interestingly, a previous report demonstrated a decrease in the number of circulating tumor cells within PyMT E2F1^−/−^ mice, suggesting a disruption to the early steps in the metastatic cascade. Other data shows remodeling of the extracellular matrix at pre-metastatic lesion sites to be important for eventual seeding of distant metastasis^21^.

Recent advances in bioinformatics methods have facilitated the investigation of cancer biology. Publicly available transcriptomic datasets have allowed for comparisons between primary tumor and distant metastatic lesions^22,23^. Next generation sequencing has furthered our understanding of cancer genomics. Studies involving the sequencing of human tumors have described the mutation rate of solid tumors^24^, and demonstrated that numerous genomic events are required for metastasis^25–27^. To determine the underlying genomic events behind altered metastatic characteristics in E2F1 KO tumors, gene expression and sequence data was analyzed. Here, we characterize the genome landscape of E2F WT and E2F1 KO tumors from both the Neu and PyMT models and uncover new targets that may be critical to tumor development and progression.

## Results

**Figure 1:**
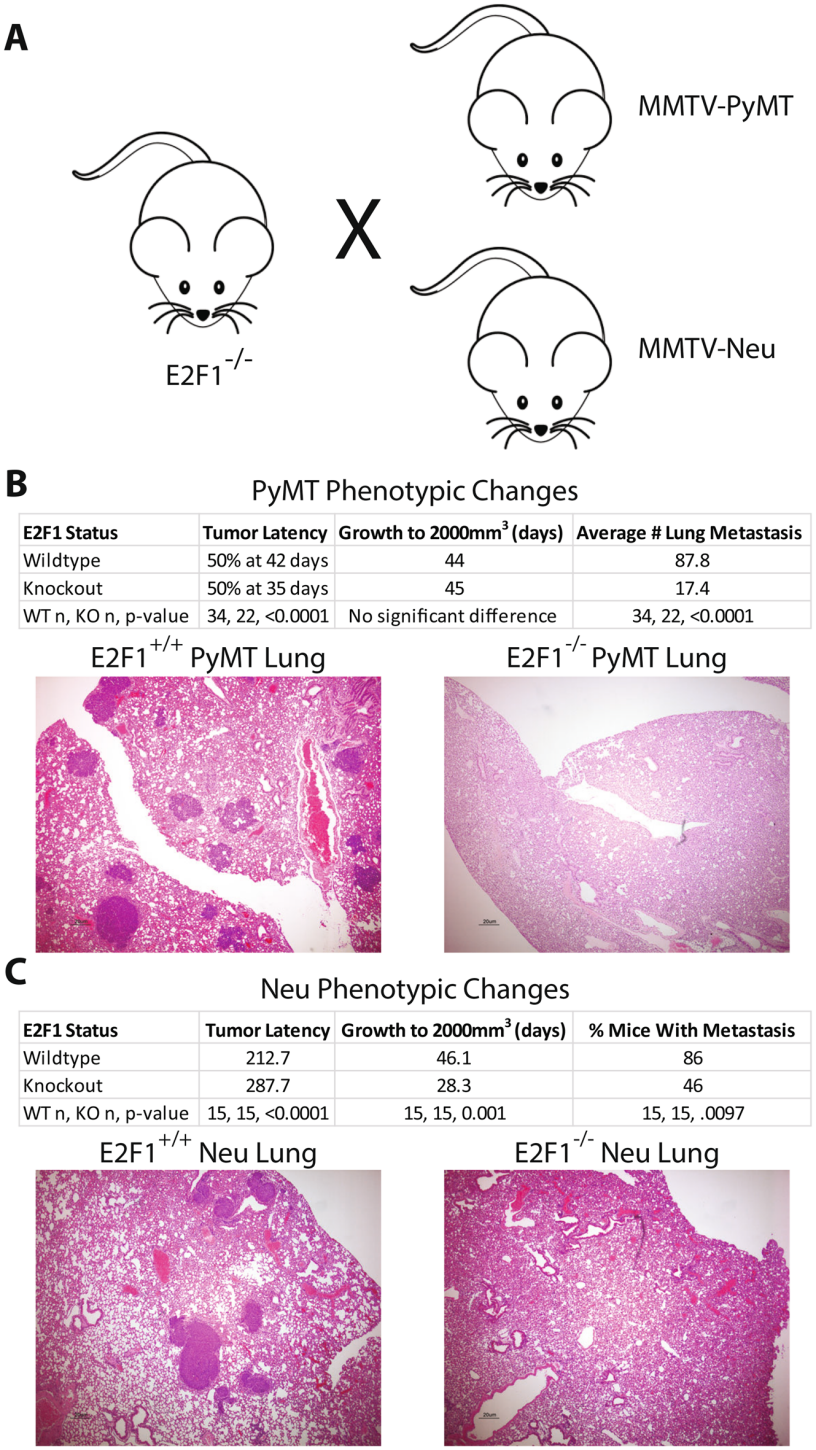
Altered phenotypic characteristics in E2F1^−/−^ tumors. (**A**) E2F1^−/−^ mice were crossed with MMTV-Neu and MMTV-PyMT mice on the FVB background to create E2F1 knockouts in both models. (**B**) Phenotypic changes seen in PyMT E2F1^−/−^ mice and (**C**) Neu E2F1^−/−^ mice, summarizing changes in latency, growth rate, and number of metastasis. H&E staining of E2F1^+/+^ mouse lung shows a large number of metastasis, while E2F1^−/−^ mice have little to no metastasis. Histology of the lungs was obtained at primary tumor endpoint.

We previously demonstrated altered phenotypic characteristics upon ablation of E2F1 within Neu and PyMT models, including changes in growth rate and tumor latency for the primary tumors (Fig. 1A). Given the short latency of PyMT mice, it was surprising to observe tumor latency in PyMT mice significantly decreased with E2F1 loss while growth rate remained unaffected. Interestingly, the opposite effect was seen within Neu E2F1^−/−^ mice, where latency was significantly increased, and growth rate was significantly increased. However, the most striking phenotype was a significant reduction of metastasis with loss of E2F1 in both strains (Fig. 1B and C).

**Figure 2:**
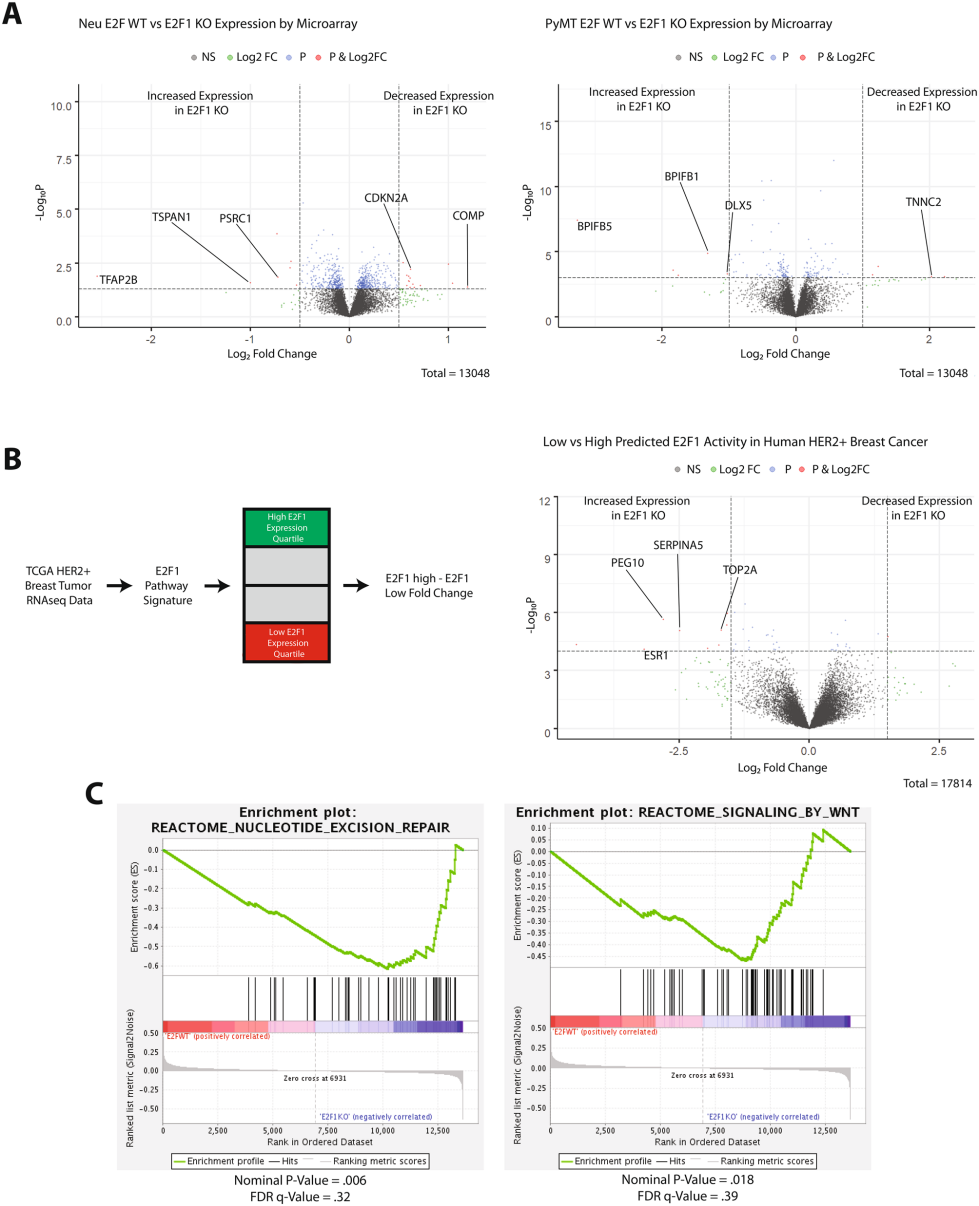
Gene expression changes in E2F1^−/−^ mouse tumors, and E2F1 low human breast cancer. (**A**) Two volcano plots show significant fold changes in genes from Neu and PyMT mouse tumors respectively. Fold change was determined by subtracting the E2F1 KO mean from the E2F1 WT mean for each gene. Fold change and *p* value cutoff for Neu tumors was .5, and .05 respectively. Fold change and *P* value for PyMT tumors was 1.0 and .001 respectively. (**B**) Diagram represents data processing steps for human TCGA data. A volcano plot shows significant fold change genes in E2F1 high versus E2F1 low human HER2+ve tumors. Fold change was determined by subtracting samples in the lowest E2F1 quartile mean from the highest E2F1 quartile mean for each gene. Fold change cutoff and *p* value for human tumors was 2.0, and 10e−60 respectively. (**C**) GSEA plots generated for E2F1 WT versus E2F1 KO tumors (Neu and PyMT combined) show enrichment of Nucleotide excision repair, and WNT signaling pathways in E2F1 KO tumors.

To determine whether gene expression differences regulated phenotypic changes in E2F1 knockout tumors, fold change differences were examined. Volcano plots revealed few genes with major gene expression changes when analyzing E2F1 WT and E2F1 KO tumors (Fig. 2A). While there were some genes with a fold change between 1 and 1.5, there were very few genes with a fold change greater than 1.5. To test whether this is recapitulated in human breast cancer, data from The Cancer Genome Atlas (TCGA) was analyzed. E2F1 activity in HER2+ve samples was determined using pathway signature analysis. Samples were stratified into quartiles for E2F1 activity and differential gene expression was determined. As shown in Fig. 2B, human breast tumors resemble mouse mammary tumors in that low E2F1 activity does not lead to vast gene expression changes. To test for genetic pathways affected by loss of E2F1, Gene Set Enrichment Analysis (GSEA) was completed on Neu and PyMT tumors with and without E2F1. GSEA analysis revealed several differentially regulated pathways, including WNT signaling, and nucleotide excision repair (Fig. 2C). Importantly, WNT signaling has been shown to regulate the epithelial to mesenchymal transition, a process involved in the metastatic cascade^28,29^.

**Figure 3:**
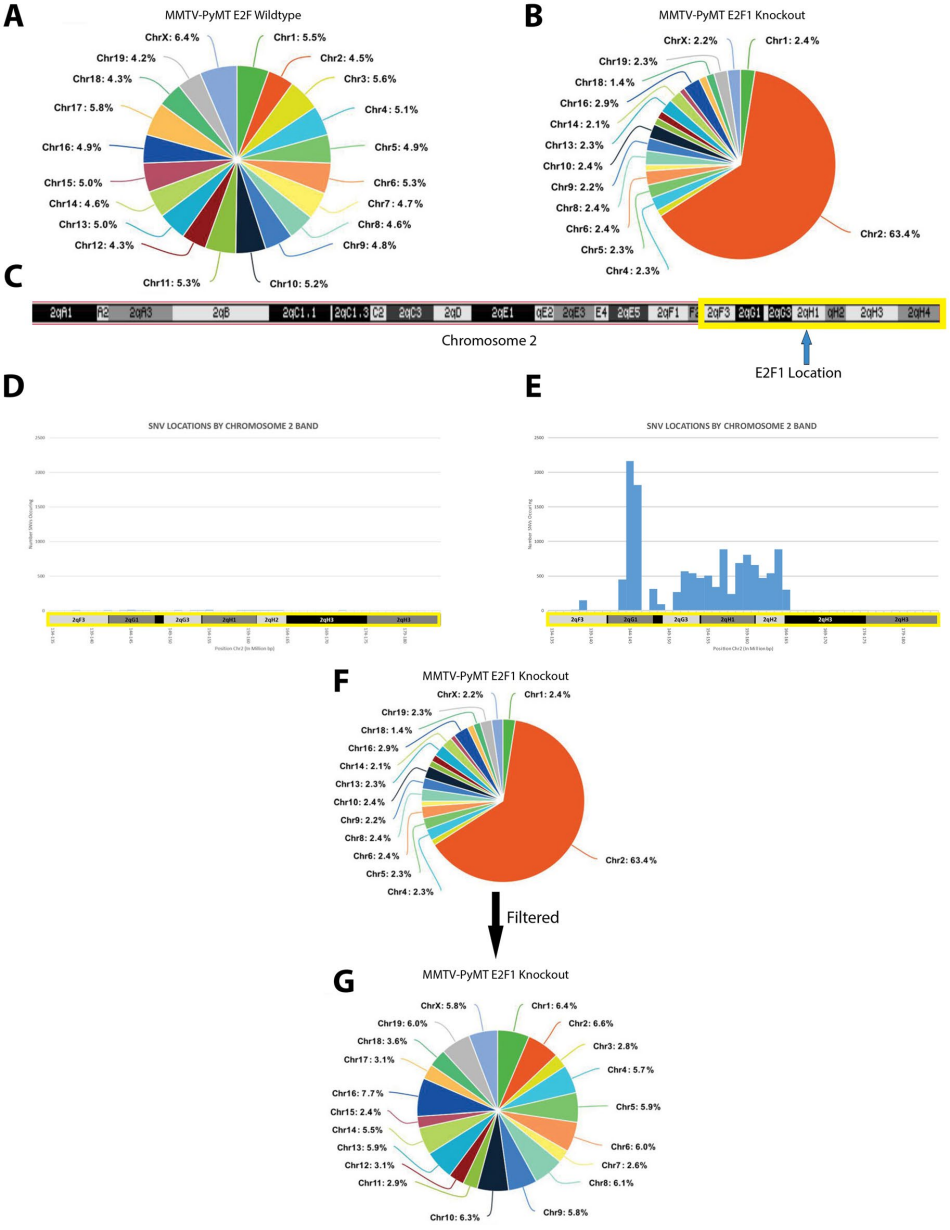
Filtering background strain to remove artifacts that have potential to confound analysis. (**A**) Pie chart from an E2F1^+/+^ PyMT tumor represents the normalized (SNVs/chromosome size) percentage of SNVs within each chromosome. (**B**) Pie chart from an E2F1^−/−^ PyMT tumor represents the normalized percentage of SNVs within each chromosome. An abundance of SNVs within chromosome 2 is observed. (**C**) The banding pattern of mouse chromosome 2. The arrow highlights the location of E2F1, and the yellow box represents the bands represented in (**D**) and (**E**). (**D**) Manhattan plot shows the number of SNVs occurring within the 2qF3–2qH3 bands of chromosome 2, in the E2F1^+/+^ sample from (**A**). (**E**) Manhattan plot shows the number of SNVs occurring within the 2qF3–2qH3 bands of chromosome 2, in the E2F1^−/−^ sample from (**B**). (**F**) Top pie chart is the same as in (**B**). Bottom pie chart represents the percentage of SNVs across each chromosome of the same sample as above, after filtering on the sv129 background.

Given that the gene expression analysis did not identify a mechanism altering metastatic potential, we examined genomic events occurring in Neu and PyMT tumors with and without E2F1. Whole genome sequencing was completed and single nucleotide variant (SNV) profiles were called for each tumor using TCGA best practices. Initial analysis of the SNV data resulted in an unexpectedly high proportion of SNVs occurring within chromosome 2 of the E2F1 knockout tumors (Fig. 3A, B). However, E2F1 is located within the qH1 band of chromosome 2 and correlated to where the increased SNVs were observed (Fig. 3C–E). While E2F1 knockout mice were backcrossed 12 generations to FVB, we hypothesized that SNV abundance was called due to residual background strain DNA from the original E2F1 knockout stain. Given that E2F1 mice were generated in the SV129 background, and Neu and PyMT mice are on the FVB background, we filtered SNV calls using a list of SNVs that were generated from comparing the SV129 background against the C57/BL6 background, the standard mouse reference genome (Fig. 3F). As a result, the majority of chromosome 2 SNV calls were filtered out, and the proportion of SNVs was roughly equal across the 19 autosomal mouse chromosomes in E2F1 WT and E2F1 KO PyMT tumors (Fig. 3G). This was also the case for E2F1 KO Neu tumors (data not shown). As such, residual background is an important caution when sequencing mouse models.

**Figure 4:**
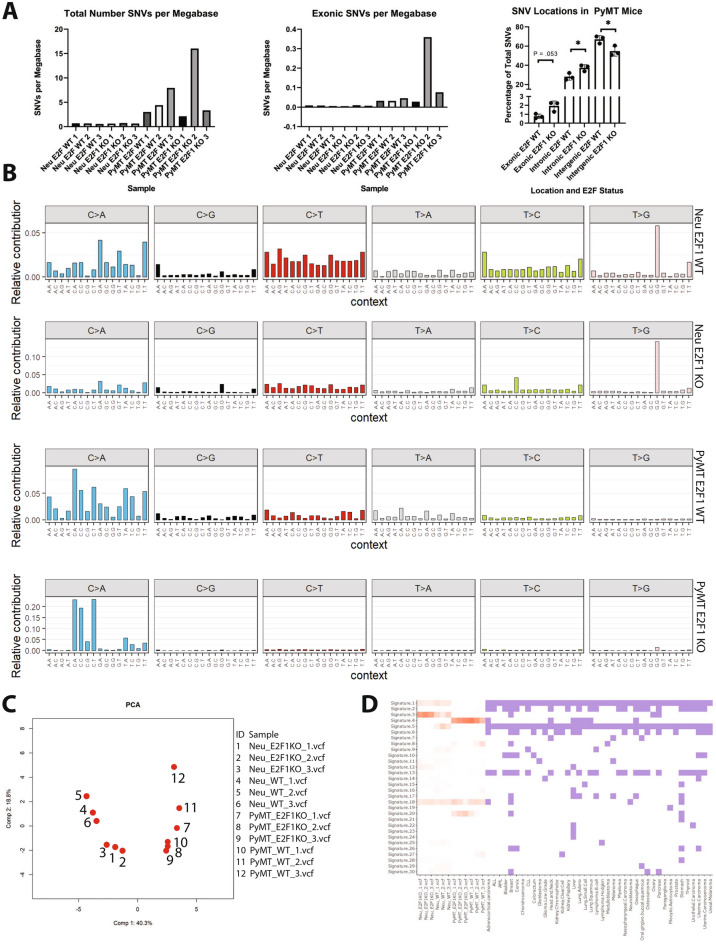
SNV mutation burden in Neu and PyMT tumors. (**A**) Bar graphs represent the number of total or exonic mutations per megabase occurring in all 12 sequenced tumors. (**B**) Shows representative mutation profiles for each of the four classes of samples sequenced. Mutation profiles are derived from 96 bp trinucleotide signatures originally developed by Alexandrov et al. Four classes of samples are Neu E2F1^+/+^, Neu E2F1^−/−^, PyMT E2F1^+/+^, PyMT E2F1^−/−^. (**C**) PCA plots derived from trinucleotide signatures show clustering of all 12 samples sequenced. (**D**) The heatmap of cancer signatures for the 12 sequenced tumors, as well as various cancers is shown.

Interestingly, the SNV mutation burden was higher in PyMT mice as compared to Neu mice (*p* value = 0.05), which was surprising due to the brief latency of PyMT tumors (Fig. 4A). Except for one PyMT E2F1 knockout tumor, the rate of exonic SNVs ranged from 0.005 to 0.08 mutations per megabase. This mutation rate is similar to previous rates shown for mouse tumors^30^, and is lower than the 1 mutation / megabase exonic mutation rate commonly observed in human breast cancer^24^.

To analyze distinct types of SNVs occurring within our tumors, and investigate potential mechanisms driving these differences, a mutation signature approach was taken^31^. While trinucleotide signatures showed similarities between Neu and PyMT tumors, there were striking differences, such as T > G mutations occurring almost exclusively in Neu tumors of either E2F1 status (Fig. 4B). The signatures for all 12 tumors are shown in Supplemental Figure 1. Principal component analysis (PCA) completed using mutation signatures from all 12 tumors shows distinct clustering between Neu and PyMT tumors (Fig. 4C). Furthermore, apart from a single E2F1 KO PyMT tumor, PCA separates E2F1 WT and E2F1 KO tumors into distinct clusters within the Neu and PyMT models. While PyMT E2F1 KO sample 2 has a sixfold increase in the number of SNVs, this is not reflected within the sample clustering of the principal component analysis. This is due to PCA being completed on the mutation signatures of the samples. For example, if sample X were to have an increased number of SNVs as compared to sample Y, but the overall mutation profile of those SNVs was similar between sample X and Y, they would cluster together.

The contribution of the 30 known COSMIC (catalog of somatic mutations in cancer) signatures to each Neu and PyMT tumor were then determined^31^. While all Neu and PyMT tumors had some contribution from signature 18, there were stark differences in other COSMIC signatures contributing to Neu and PyMT tumors (Fig. 4D). For example, Neu tumors had contributions from signatures 1 and 3, while PyMT tumors were associated with signatures 4 and 20. Furthermore, there were signature differences when comparing E2F1 WT tumors to E2F1 KO tumors within the Neu and PyMT models. For example, Neu E2F1 WT tumors were associated with signatures 5 and 9, while Neu E2F1 KO tumors lacked these associations. Neu E2F1 KO tumors also had an association with signature 12, while Neu E2F WT tumors lacked this signature. When analyzing the proposed etiology for these signatures, Neu tumor signatures are associated with age, while PyMT tumor signatures have no age association, which correlates with Neu and PyMT tumor latency (Supplemental Figure 2). Interestingly, Neu tumors also have an association with inefficient double stranded break repair (DSB), with E2F1 KO tumors being more highly associated than E2F1 WT tumors. E2F1 has been found to recruit DSB processing factors, particularly NBS1, to DSB sites, which serves as a possible explanation for this signature^32^. PyMT E2F1 KO tumor signatures were not associated with DSB, but were highly associated with the smoking signature number 4, and defective DNA mismatch repair (MMR) signature 20. While it may seem counterintuitive that PyMT E2F1 KO tumors would be associated with one MMR signature and not the others (numbers 6, 15, and 26), it is entirely possible for this to occur. Multiple mutational profiles can be associated with a particular etiology, even though the mutational profiles themselves are distinct from each other.Together, these data suggest E2F1 loss drives differences in DNA repair and tumor etiology.

**Figure 5:**
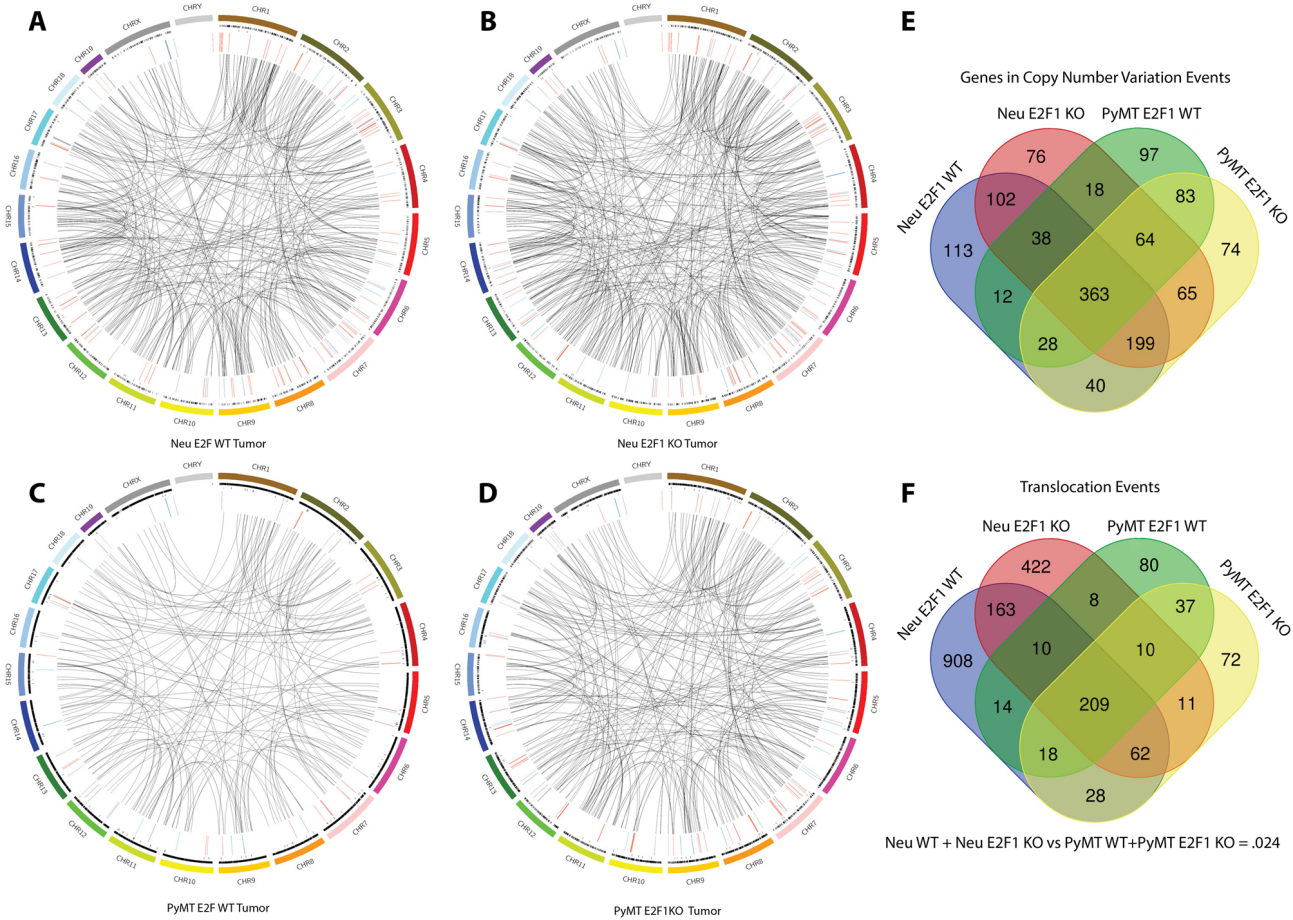
Mutation burden in Neu and PyMT tumors. (**A**) Circos plot for a representative Neu E2F1^+/+^ sample. (**B**) Circos plot for a representative Neu E2F1^−/−^ sample. (**C**) Circos plot for a representative PyMT E2F1^+/+^ sample. (**D**) Circos plot for a representative PyMT E2F1^−/−^ sample. For (**A**)–(**D**) Circos plots, outer most ring represents the mouse chromosomes. Four successive inner rings represent the following mutation types; total SNVs, exonic SNVs, Copy number variation with green being amplification and red being deletion, and translocations. Circos version .69-6 was used to generate the plots and is available at www.circos.ca. (**E**) Venn diagram showing the overlap of genes within copy number events. Consensus copy number events were generated for each of the three samples within the four sample classes. Genes were then extracted and compared across the sample classes. (**F**). Venn diagram showing the overlap of translocations occurring within the four sample classes. Consensus translocations calls from each of the three samples within each class were generated, and the four classes were then compared.

Multiple programs were also used to determine copy number variants and translocations occurring within Neu and PyMT tumors (Fig. 5A–D). Based on consensus CNV calls from two programs, over 98% of the copy number events were small in size (under 1 mb), while relatively few larger events (above 1 mb) were observed. Surprisingly, there was a large amount of copy number gene overlap between the E2F WT and E2F1 KO tumors (Fig. 5E). The large number of shared genes involved in copy number events may indicate E2F1 loss is not a primary driver of these events.

**Figure 6:**
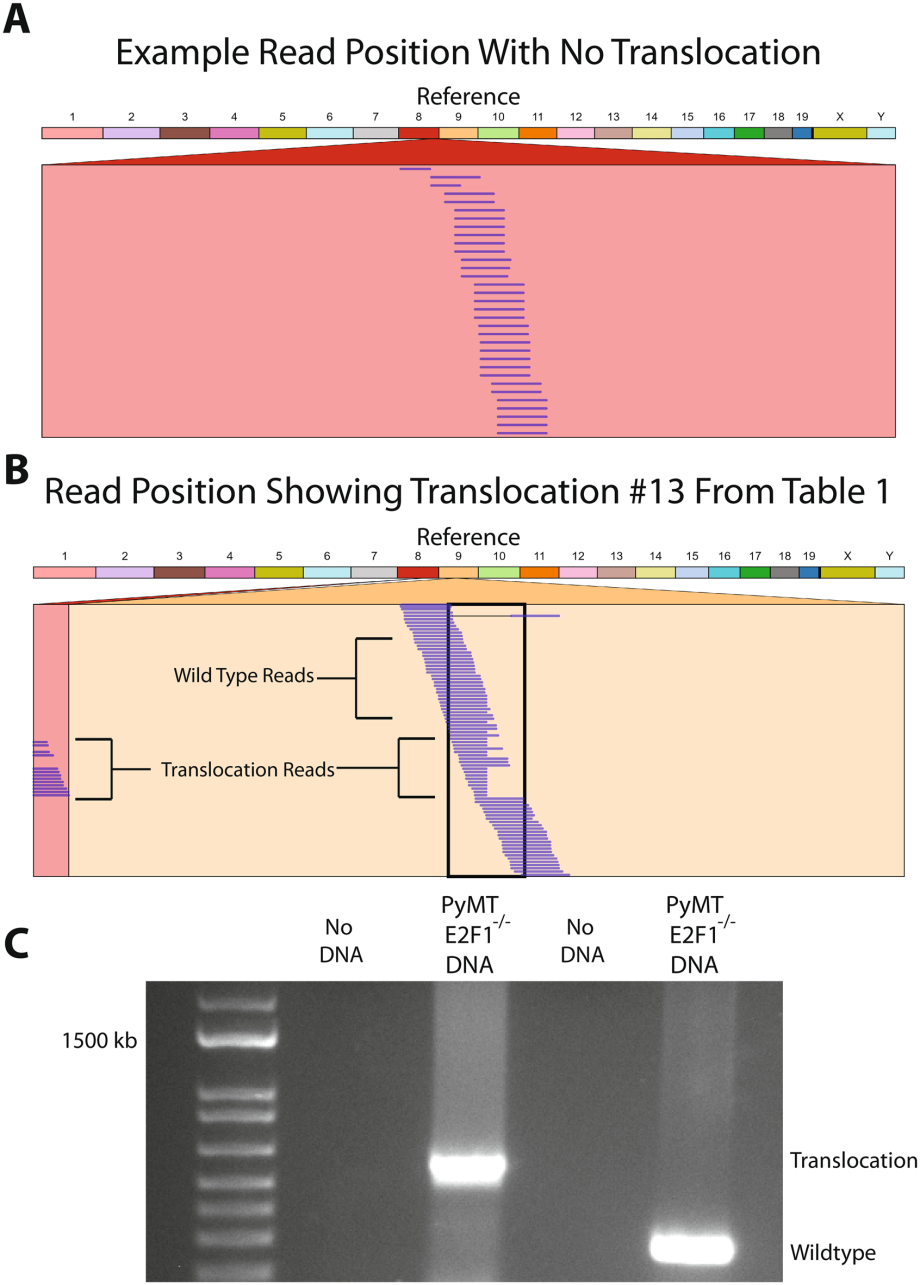
Verification of translocation calls. (**A**) Example of a GenomeRibbon plot where no structural variation occurs. The top colored bands represent each chromosome of the mouse, and the red box below represents the location searched within a sample’s bam file. Each line within that box represents a different read. (**B**) A GenomeRibbon plot representing translocation number 13 from Table 1. Translocated reads are shown between chromosome 9 and chromosome 8. (**C**) Gel image of the chromosome 8/9 translocation from the GenomeRibbon plot above. DNA was from a PyMT E2F1^−/−^ tumor. Both translocation and wild type tumor DNA were amplified. Translocated reads were amplified using a primer set flanking the region where the two translocated ends ligate. Gel image is cropped to remove excess, see supplemental for full gel.

There were also a surprisingly large number of translocations occurring within the Neu and PyMT tumors. When comparing average number of translocations per sample across the genomic models, there were statistically more translocations occurring within Neu tumors than PyMT tumors, regardless of E2F1 status. When comparing E2F1 status within each model, there was no statistically significant difference (Fig. 5F). To confirm the translocation calls made by Delly and Lumpy, 20 translocations from each tumor were chosen at random and read evidence for these translocations was analyzed using Genome Ribbon^43^. Translocation read data for one tumor is shown in Table 1. All tumors had at least 75% of translocations with some read support, with 9 of 12 tumors having at least 85% of translocations with some read support (Supplemental Table 1). Interestingly, all translocation events analyzed had a varying level of wild type reads present. Since care was taken to exclude normal tissue when primary tumor was collected for sequencing, and since the abundance of wild type reads is fairly large for many of the translocation sites, this suggests a large amount of heterogeneity within the tumors. While some normal tissue (vasculature, immune etc.) is present in any tumor, the prevalence of wild type reads is far below that observed for mutations. To verify one of the translocation events from Table 1, PCR was completed with primers flanking the translocation junction. Both translocated and wild type reads were present at the breakpoint, confirming the existence of the translocation (Fig. 6). Based on this evidence, upwards of 80% of the translocations were predicted to be real events.

**Table 1:** Supporting reads for 20 randomly selected translocations from the tumor in Fig. 6.

Translocation #	Position 1	Position 2	Supporting reads (approximate)	Total reads (exact)	% Support
1	3_65552053	2_20941004	10	61	16.39
2	2_161669843	18_78292047	10	119	8.40
3	15_43944218	1_112318855	6	89	6.74
4	13_23307876	11_88303305	11	104	10.58
5	5_5609182	18_56166475	14	77	18.18
6	5_7058436	2_8931319	4	49	8.16
7	16_83532604	14_96841358	15	83	18.07
8	3_153288050	17_10818207	15	88	17.05
9	16_83532819	14_96841377	19	94	20.21
10	16_18368004	12_80665928	10	85	11.76
11	8_102861241	17_67932443	0	57	0.00
12	14_21312516	11_9863013	16	234	6.84
13	9_55224433	8_85188141	13	82	15.85
14	X_38480149	9_55983052	12	70	17.14
15	6_73162349	16_96121815	3	34	8.82
16	4_43262573	3_113857214	4	68	5.88
17	5_62573934	13_86796353	2	77	2.60
18	3_135929183	1_139635092	9	95	9.47
19	6_67680744	4_147419479	0	158	0.00
20	7_79199005	19_40536086	14	81	17.28

Random translocations were selected by inputting all translocations from the tumor into Excel, and using the RAND() function to assign a random number.

The 20 highest translocations were then selected.

Positions 1 and 2 represent the translocation breakpoint.

Genome Ribbon was used to analyze read evidence.

To determine whether cancer and metastasis related genes were mutated within E2F1 WT and E2F1 KO tumors, the mutation list was filtered with known cancer genes from COSMIC. This analysis found mutations in a number of cancer associated genes (Supplemental Table 2). While a few of the genes listed in Supplemental Table 2 have known metastatic implications, they were not consistently mutated within the sample groups, or were mutated exclusively within E2F wildtype tumors. To identify whether an abundance of mutations occurred within particular pathways comparing E2F1 knockout to wildtype tumors, a database mining approach was taken using Gather^33^. First, genes with potentially impactful mutations were stratified into two gene lists that were distinct in E2F1^−/−^ and E2F1^+/+^ tumors. Potentially impactful mutations included SNVs causing stop gain or nonsynonymous mutations, translocations causing truncated or fusion genes, and copy number segments resulting in the amplification or deletion of genes. These two gene lists were then applied to Gather to determine whether Gene Ontology (GO) lists or KEGG pathways were significantly mutated. This analysis determined a number of significant GO lists that were present within the gene list from E2F1^−/−^ tumors, but not E2F1^+/+^ tumors.

**Figure 7:**
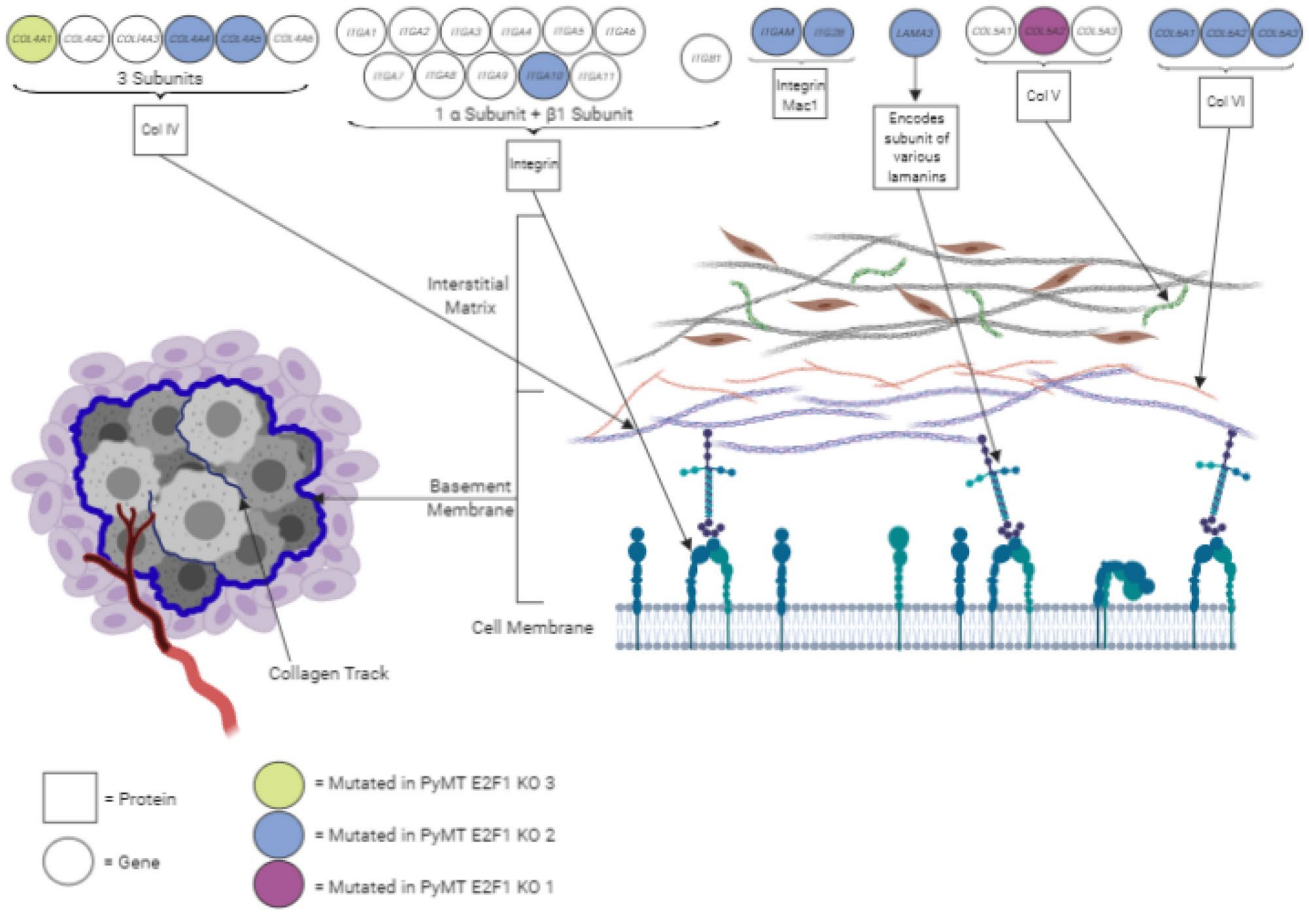
Mutations in basement membrane genes. Diagram shows various mutations occurring in genes that code for proteins making up the basement membrane and interstitial matrix. Circles at top indicate genes with colors representing 1 of 3 sequenced E2F1^−/−^ PyMT tumors that has a mutation in that gene. Image on left represents a breast tumor with surrounding basement membrane. Image on right represents the basement membrane and interstitial matrix on the outer edge of a tumor.

In fact, the top three GO pathways associated with E2F1 KO tumors were involved in cell adhesion (GO:0007155 *p* value ≤ 0.0001, GO:0007156 *p* value ≤ 0.0001, GO:0016337 *p* value = 0.0001). Genes in those cell adhesion GO annotations included various collagens, integrins, and cadherins (Fig. 7). Previous research has shown collagens to be important for tumor maintenance, angiogenesis, and metastasis^18^. Collagen IV is the major component of the basement membrane and is comprised of heterogeneous trimers stemming from six COL4A genes. Three collagen IV genes were found mutated in different PyMT E2F1 KO tumors. Other mutations within PyMT E2F1 KO tumors include COL5A2, with collagen V being a component of the interstitial matrix, COL6A1-3, with collagen VI being abundant in the tumor invasive front^21–23^ and several integrin and cadherin genes. Interestingly, a closer examination of the gene expression data revealed the integrin pathway was also found to be upregulated within E2F WT tumors, but not E2F1 KO tumors (Supplemental Tables 3, 4) . There was also an abundance of intronic and synonymous mutations within these genes, suggesting they may be hypermutated due to the disruption of E2F1 within the model, although this hasn’t been statistically verified. Indeed, of the 64 mutated genes within the cell adhesion Gene Ontology number 0007155, half were noted to have an E2F1 binding motif using TRANSFAC (*p* value = 0.003, data not shown). With E2F1 known to regulate the cell cycle as well as a number of genes involved in DNA repair and adhesion, it is feasible that loss of E2F1 could result in an abundance of mutations within certain gene profiles through a disruption of the cell’s ability to undergo DNA repair during the S phase. E2F1 loss and corresponding disruptions to the cell cycle, especially during S phase could conceivably lead to an increased mutation burden, potentially within E2F regulated genes. E2F1 has also been shown to recruit nucleotide excision repair and double stranded break repair factors to sites of DNA damage^32,34,35^. It is possible that loss of recruitment of these factors could lead to inefficient DNA repair, and an increased mutational burden, although this would need to be further explored.

## Discussion

Ablation of E2F1 in PyMT and Neu transgenic mice results in a significant decrease in pulmonary metastasis. To determine whether gene expression changes were responsible for altered phenotypes, transcriptomic data was analyzed but showed no large changes in gene expression between E2F1^+/+^ and E2F1^−/−^ tumors. This was recapitulated in human HER2+ breast cancers after separation into E2F1 high/low quartiles. GSEA revealed several pathways differentially regulated between E2F1^+/+^ and E2F1^−/−^ tumors, but without obvious implications in regulating metastasis. To test for genomic alterations impacting metastasis, we completed WGS of E2F1^+/+^ and E2F1^−/−^ tumors in Neu and PyMT models. Mutation trinucleotide signatures showed differences between etiology of Neu and PyMT tumors, as well as between the E2F1 knockout and WT tumors. Neu tumors were more closely associated with double stranded break repair, while PyMT tumors were associated with DNA Mismatch Repair. As noted, Neu E2F1 KO tumors were more closely associated with defective double stranded break repair than Neu E2F wildtype tumors. An interesting question that warrants further investigation would be whether this was due to increased alterations within these genes upon loss of E2F1, or due to some other transcriptional function of E2F1. Analyzing mutated genes for GO and KEGG pathways revealed alterations in cell adhesion. Further analysis of these genes uncovered a role in the basement membrane and interstitial matrix, which could be a potential mechanism for disruption of the metastatic cascade.

Sequencing data from genetically engineered mouse models is largely lacking, with only a few models having been sequenced^30,36–38^. SNV mutation rates between previous studies and ours indicate similarities, and small discrepancies may be explained through differences in data processing methods. For copy number variation prior research has shown numerous small copy number events and a few larger events^36^, although this was estimated from whole exome sequencing data. This was recapitulated in our data, with the exception that large events were not prevalent after taking the consensus of two structural variant callers. We also noted a substantially greater number of translocations within the mouse tumors as compared to a previous study comparing Neu and PyMT wildtype tumors, while the same trend of Neu tumors having more translocations than PyMT tumors held. This increase in called translocations is likely due to differences in calling methods. Overall, the field would benefit from a large comparison of mouse tumor sequencing data with tumors analyzed under the same parameters.

After analyzing mutated genes using a pathway approach, many genes involved in cell adhesion were found having potentially impactful mutations in E2F1 knockout tumors, but not E2F1 wild type tumors, including various collagens, integrins and cadherins. Of the mutated genes found important to cell adhesion, genes such as *Col4a1* are important components of the basement membrane and are involved in tumor progression. Disruptions to the basement membrane and collagen formation has potential to disrupt the metastatic process. This theory is supported by previous data we generated, which found a significant decrease in circulating tumor cells^7^. Interestingly, we have also previously noted amplification of *Col1a1* in Neu E2F1 WT tumors which impacted the metastatic process^28^. Combined, these data suggest collagens and proteins within the basement membrane are important to the metastatic process in Neu and PyMT tumors.

SNV profiling for human tumors has utility for both discovery and treatment purposes. Sequencing of human breast tumors has revealed larger genomic trends as well as mutation rates for oncogenes and tumor suppressors^39^. The importance of determining SNVs within mouse models is evidenced by previous research from our lab and others^30,36^. Potential sources of error when determining SNVs can stem from differing genetic background within mice, even after backcrossing, as well as being too loose or too stringent with the filtering process. Interestingly, our prior work identified and validated a SNV in *Ptprh* in PyMT tumors^30^, but this mutation was not present within this sequence analysis. While the initial paper stipulated an SNV call must pass 3 of 4 SNV calling programs, the work herein stipulated a call must pass 3 of 3 programs used, leading to the discrepancy. When analyzing the SNV data for each program used, a *Ptprh* SNV was called from SomaticSniper and Varscan, but not called from Mutect2. This suggests the usage of multiple programs to call SNVs is more applicable for discovery purposes, and that less stringent filtering parameters may be beneficial.

When analyzing copy number alterations and translocations within the models, there were a surprising lack of differences across E2F1 status, suggesting E2F1 loss is not a primary driver of these events. Furthermore, the varying read support seen for confirmed translocations indicates a high amount of tumor heterogeneity occurring in both models, regardless of E2F1 status. While there were numerous COSMIC associated genes mutated within the models, no mutations conserved between E2F1 knockout tumors (within or across models) were immediately apparent as important to the metastatic process.

Analyzing gene expression changes between E2F1 WT and E2F1 KO tumors showed no major changes upon E2F1 loss. This was recapitulated among human HER2+ve breast cancer tumors stratified between low and high E2F1 activity. The lack of large gene expression changes may indicate that numerous small changes result in phenotypic alterations, or that genomic mutations are leading to altered protein function/localization. Interestingly, the gene encoding Transcription Factor AP-2 Beta was significantly upregulated in Neu E2F1 KO mice. This, combined with the data showing a lack of major gene expression changes between E2F1 WT and E2F1 KO tumors, indicates some possible compensation by Transcription Factor AP-2 Beta, as well as other members of the E2F family^5,8^. The sequencing data from E2F1^−/−^ Neu and PyMT mice indicate phenotypic changes may be due to an abundance of mutations in particular pathways, in addition to minor expression changes. Taking into consideration that the metastatic process likely originates from a small population of metastatic cells within the primary tumor, the contribution of a few metastatic cells to the bulk tumor gene expression or sequencing data may cause key events to be lost within the noise of the primary tumor. Future work will address these issues through single cell sequencing and gene expression in matched primary and metastatic tumors.

## Methods

### Animal models

No live animals were directly used for this study. Gene expression and sequencing data was obtained previously^6,8,28^. In those studies, all methods involving animals were carried out in accordance with relevant guidelines and regulations. All experimental protocols involving animals in the previous studies were approved through Michigan State University.

### Gene expression analysis

Gene expression data was described previously^6,8^. Volcano plots for Neu and PyMT tumors were generated by removing outliers for each sample group using Nowaclean (Holsb, Einar. 2017. “nowaclean”), samples greater than 3.0 standard deviations away when constructing PCA plots were removed. Data were log2 transformed, and the mean for each gene was calculated within the four sample groups. Fold change was calculated by subtracting the E2F1 KO mean from the E2F1 WT mean for each gene. *P* values were calculated and data plotted using EnhancedVolcano (Blighe, Kevin. 2018. “EnhancedVolcano”) in R. Human RSEM normalized RNAseq breast cancer data from TCGA was downloaded from UCSC Xena, filtered to HER2+ samples, and sorted by E2F1 expression. Lower and upper quartiles were kept and data were processed for volcano plots as above. GSEA plots were generated from combining Neu and PyMT gene expression datasets. Datasets were collapsed and combatted to remove batch effects. GSEA was run using GenePattern^40^.

### Whole genome sequencing and processing

Raw whole genome sequencing data from mouse tumors was previously obtained^28^. Briefly, three samples from each group (total of 12) were used, DNA from flash frozen extracted following manufacture’s protocol for Qiagen Genomic-tip 20/G kit. Sequencing was completed at a depth of 40 × with paired end, 150 base pair reads. DNA was prepared and sequenced using Illumina TruSeq Nano DNA library preparation and an Illumina HiSeq 2500. For this study, raw fastq files were assessed for quality control using FASTQC (https://www.bioinformatics.babraham.ac.uk/projects/fastqc/) and trimmed using Trimmomatic^41^. Files were aligned to mm10 mouse reference using BWA MEM^42^ with standard parameters. Picard tools (“Picard Toolkit.” 2019) was used to add read groups and remove duplicates. Samtools^43^ was used to sort and index files.

### Variant calling

Somatic SNVs were called using SomaticSniper^44^, Mutect2^45^, and VarScan^46^. Consensus calls were merged using R (R Core Team (2018)) base programming, and mutations were only kept if called by all three programs. SNV calls were filtered using base R to account for differences between the FVB strain and mm10 alignment (C57/BL6), as well as differences between the SV129 strain (original E2F1 mouse background) and C57/BL6. SNVs were annotated using Annovar^47^. CNVs were determined by keeping the consensus of Lumpy^48^ and Delly^49^. Consensus was determined using Intansv (Yao W 2019) at a threshold of 0.2, and events smaller than 10,000 bp were filtered out. Intansv was also used to annotate CNV events. Translocations were called using Lumpy and Delly, and filtered based on read evidence. Lumpy calls were kept if they had at least 20 supporting split end and paired end reads, Delly calls were kept if there was split end and paired end read evidence for the call. WT FVB mouse sequence was used as a normal control.

### Mutation signatures

Trinucleotide mutation signatures were completed using the Musica^50^ shiny app in R. Musica code was altered to allow for the use of the mouse mm10 reference genome.

### Circos plots

Circos plots were generated for each sample using CIRCOS version 0.69^51^. Genetic variants were plotted according to the mm10 reference genome.

### Translocation verification

Read evidence for 20 randomly selected translocations from all 12 sequenced samples was examined using GenomeRibbon^52^. For PCR verification, primers were designed with at least 400 bp flanking the predicted breakpoint.

## Supplementary Information

Supplementary Information.
