# Supplementary material for: Metastasis is altered through multiple processes regulated by the E2F1 transcription factor
Source: Sci Rep. 2021 May 4;11:9502. doi: 10.1038/s41598-021-88924-y (PMC8097008; doi:10.1038/s41598-021-88924-y)
Supplement: Supplementary file 1 — Supplementary Information. [file 41598_2021_88924_MOESM1_ESM.pdf]

Supplementary Information

Metastasis is altered through multiple processes regulated by the E2F1 transcription factor

Matthew R. Swiatnicki and Eran R. Andrechek

Supplemental Figure 1

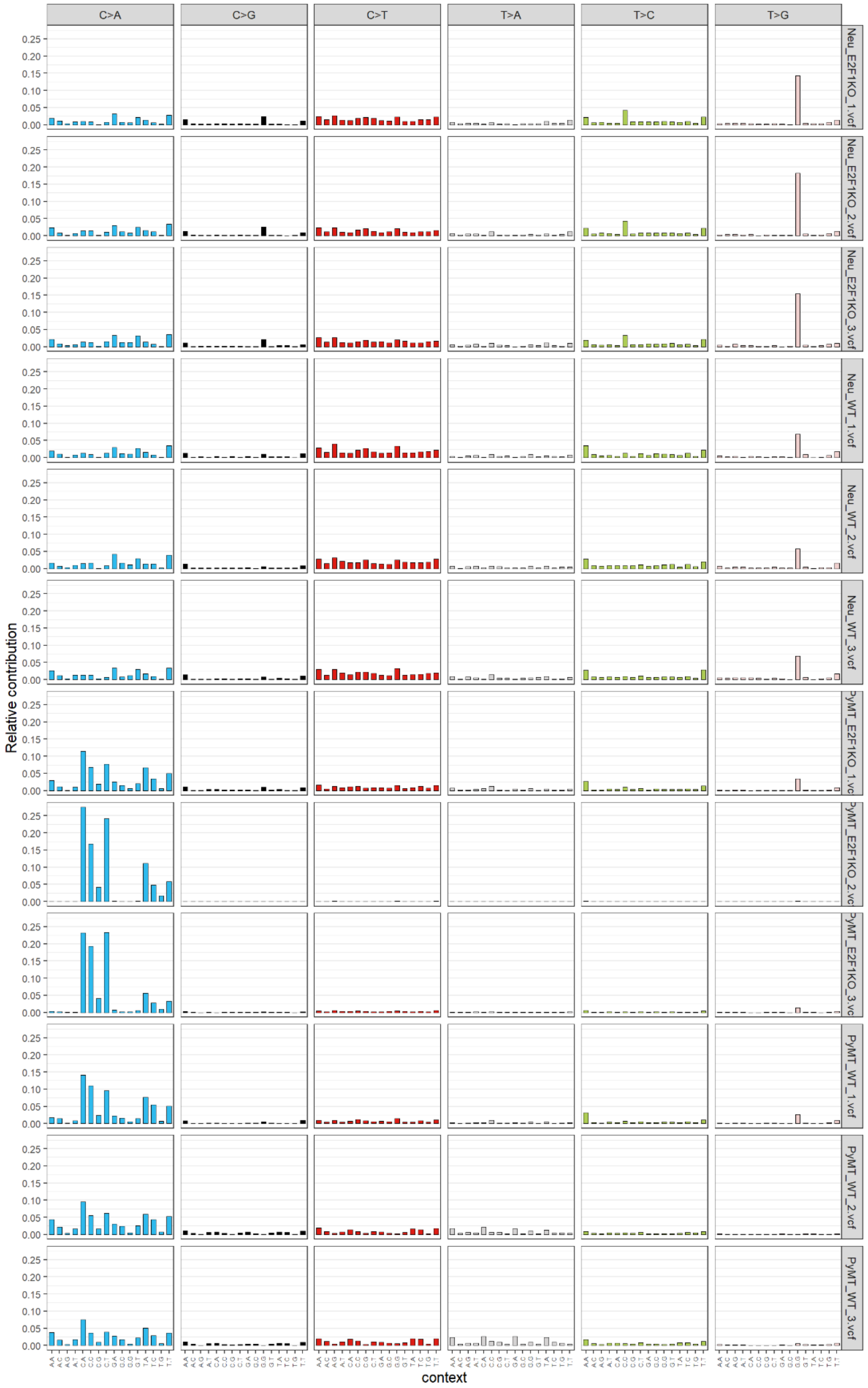

Supplemental Figure 1: Mutation profiles for all 12 Neu and PyMT mouse tumors corresponding to four classes in Figure 4B. Mutation profiles derived from 96 bp trinucleotide signatures originally developed by Alexandrov et. al.

Supplemental Figure 2

Signature	Proposed_Etiology	Neu_E2F1KO_1	Neu_E2F1KO_2	Neu_E2F1KO_3	Neu_WT_1	Neu_WT_2	Neu_WT_3	PyMT_E2F1KO_1	PyMT_E2F1KO_2	PyMT_E2F1KO_3	PyMT_WT_1	PyMT_WT_2	PyMT_WT_3
1	Age	0.148	0.117	0.111	0.185	0.128	0.14	0	0	0	0	0	0
2	APOBEC	0	0	0	0.004	0.014	0.002	0.004	0	0	0	0.019	0.022
3	BRCA1 / BRCA2 (failure of DNA DSBR / large INDELs)	0.518	0.59	0.506	0.254	0.138	0.274	0	0	0	0	0	0
4	Smoking	0	0	0	0	0	0	0.471	0.557	0.616	0.65	0.558	0.368
5	Unknown (all cancer types)	0	0	0	0.107	0.277	0.176	0	0	0	0	0	0
6	Defective DNA MMR / MSI (small INDELs)	0	0	0	0	0	0	0.003	0	0	0	0	0
7	UV light	0	0	0	0	0	0	0	0	0	0	0	0.004
8	Unknown (breast cancer and medulloblastoma)	0	0	0	0	0	0.009	0.074	0	0	0	0.082	0.201
9	POLH (CLL, BCL)	0.012	0	0.004	0.052	0.039	0.07	0.017	0	0	0	0	0.03
10	POLE (ultra-hypermutation)	0.002	0.003	0.009	0.01	0.015	0.006	0	0	0	0	0	0
11	Alkylating agents	0.048	0.02	0	0.038	0.033	0.029	0	0	0	0	0	0.022
12	Unknown (liver cancer)	0.095	0.066	0.047	0.018	0.01	0.003	0	0	0	0	0	0.02
13	APOBEC	0	0	0	0	0	0	0.009	0	0	0.003	0.012	0.01
14	Unknown (uterine cancer and glioma / hypermutation)	0	0	0.032	0.023	0	0	0.001	0	0	0	0	0
15	Defective DNA MMR (small INDELs)	0.004	0.022	0.01	0.02	0.007	0.022	0	0	0	0	0	0
16	Unknown (liver cancer)	0	0	0	0.053	0	0	0	0	0	0	0	0.046
17	Unknown (different cancers)	0	0	0	0	0	0	0	0	0	0	0	0
18	Unknown (different cancers)	0.133	0.163	0.178	0.129	0.17	0.147	0.263	0.192	0.085	0.2	0.227	0.177
19	Unknown (pilocytic astrocytoma)	0	0	0.009	0	0	0.005	0	0	0	0	0	0
20	Defective DNA MMR (small INDELs)	0	0	0	0	0	0	0.154	0.251	0.299	0.144	0.051	0.006
21	Unknown (stomach cancer / MSI)	0	0	0	0	0.001	0	0	0	0	0	0	0
22	Aristolochic acid	0	0	0	0	0	0	0	0	0	0	0	0
23	Unknown (liver cancer)	0	0	0	0	0	0	0	0	0	0	0	0
24	Aflatoxin	0	0	0	0	0	0	0	0	0	0	0	0
25	Unknown (Hodgkin lymphoma)	0	0	0.029	0.032	0.045	0.026	0	0	0	0	0	0
26	Defective DNA MMR (small INDELs)	0	0	0	0	0	0	0	0	0	0	0	0
27	Unknown (kidney clear cell carcinomas / small INDELs)	0	0	0	0	0	0	0	0	0	0	0.05	0.082
28	Unknown (stomach cancer)	0.027	0.019	0.012	0.015	0.018	0.011	0.004	0	0	0.003	0	0
29	Tobacco chewing	0	0	0	0.034	0.055	0.035	0	0	0	0	0	0
30	Unknown (breast cancer)	0.014	0	0.053	0.025	0.049	0.046	0	0	0	0	0	0.011

Supplemental Figure 2: Table showing contribution of each proposed tumor etiology for each of the 12 mouse tumors. Numbers represent a proportion of the whole

Supplemental Figure 3

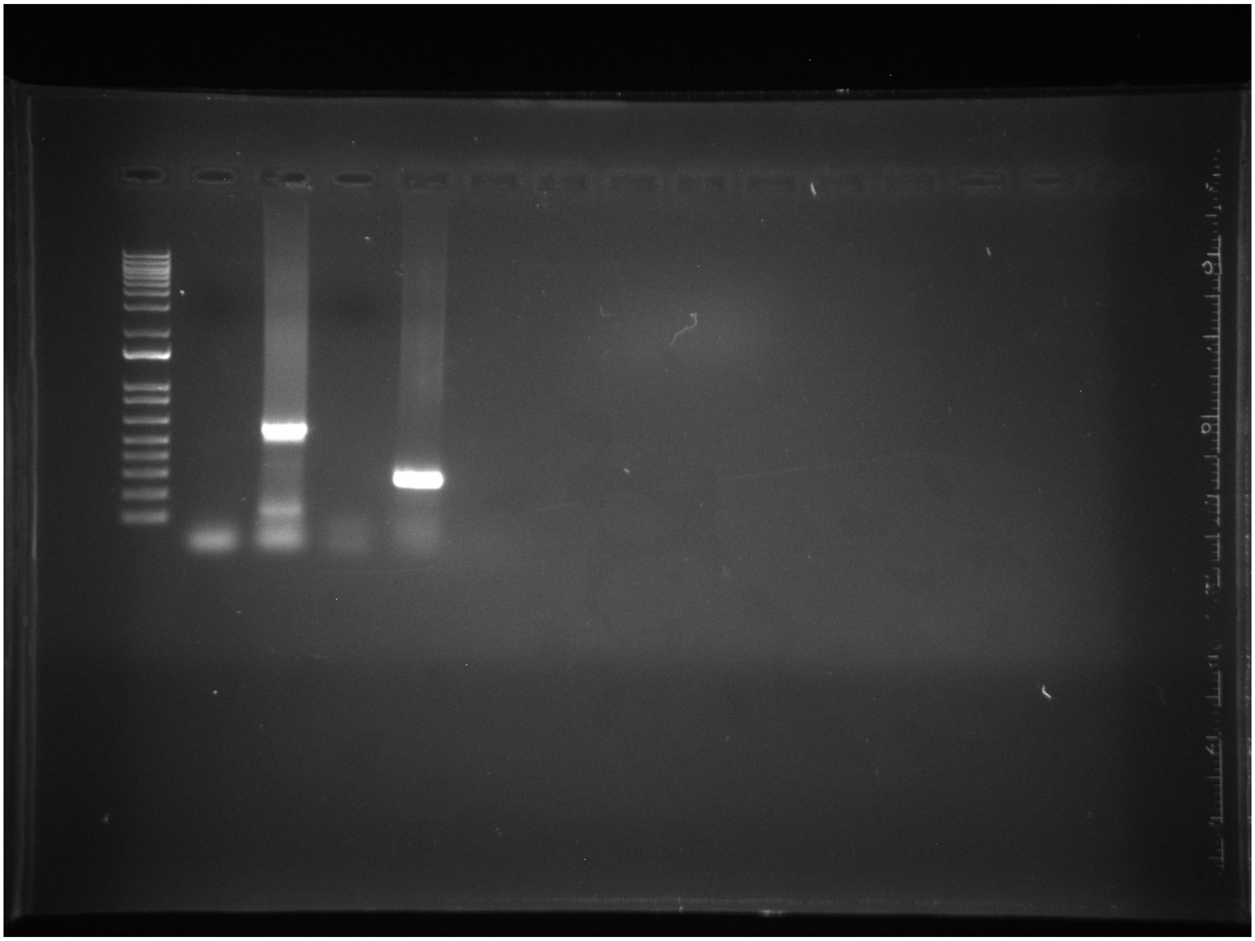

Supplemental Figure 3: PCR gel electrophoresis image showing un-cropped version of gel in figure 6.

Supplementary Table 1

Tumor	Translocations with Extensive* Read Support	Translocations with Low* Read Support	Translocations with no Read Support	% Extensive Support	% with at Least Some Support	Average Read Support (%)
Neu_E2F1KO_1	18	1	1	90	95	14.5
Neu_E2F1KO_2	17	0	3	85	85	14.78
Neu_E2F1KO_3	13	2	5	65	75	8.82
Neu_WT_1	17	1	2	85	90	15.83
Neu_WT_2	13	3	4	65	80	10.22
Neu_WT_3	18	1	1	90	95	12.42
PyMT_E2F1KO_1	16	2	2	80	90	13.21
PyMT_E2F1KO_2	18	1	1	90	95	13.48
PyMT_E2F1KO_3	17	1	2	85	90	10.8
PyMT_WT_1	14	4	2	70	90	13.62
PyMT_WT_2	13	3	5	65	80	14.94
PyMT_WT_3	15	4	1	75	95	14.06

* Extensive read support is deemed greater than 5% of reads supporting the translocation
* Low read support is deemed greater than 0, but less than 5% of reads supporting the translocation

Supplemental Table 1: Table showing read support for 20 randomly drawn translocations within each of the 12 mouse tumors. To pick 20 random translocations, for each tumor, all translocation events were imported into excel and a random number was assigned using RAND() function. These were then sorted highest to lowest, and the 20 highest translocations were taken. Translocation read support was analyzed using GenomeRibbon.

Supplemental Table 2

Cosmic Cancer Genes Exclusive to E2F1 KO Tumors	Mutation Type	Cosmic Cancer Gene Mutations Exclusive to E2F1 WT Tumors	Mutation Type
ABL1	Downstream	AFF4	Exonic Nonsynonymous SNV
AFF1	Exonic Nonsynonymous SNV	ATP1A1	UTR3
AKT2	UTR3	BAP1	Exonic Nonsynonymous SNV
ALK	Exonic Synonymous SNV	BCL2	UTR3
ANK1	Exonic Nonsynonymous SNV	BCL7A	UTR3
AR	Exonic Nonsynonymous SNV	CARD11	Downstream
AR	UTR3	CASP3	Upstream
ARHGEF10	Upstream	CHEK2	Exonic Synonymous SNV
ARID1A	Exonic Nonsynonymous SNV	CPEB3	UTR3
ATM	Exonic Nonsynonymous SNV	CTNNB1	Exonic Nonsynonymous SNV
ATRX	UTR3	ETV4	Downstream
AXIN1	Exonic Nonsynonymous SNV	FLI1	UTR3
BAZ1A	Translocation Gene Fusion	FOXO4	UTR3
BCL11A	Exonic Nonsynonymous SNV	LHFP	Translocation transcript ablation
BCL9	Exonic Synonymous SNV	LMNA	Exonic Nonsynonymous SNV
BCL9L	Exonic Synonymous SNV	MSH2	Exonic Stopgain
BRD4	Downstream	NRG1	Exonic Synonymous SNV
CAMTA1	UTR3	PIK3R1	UTR3
CASP9	UTR3	PLAG1	Exonic Nonsynonymous SNV
CBLB	UTR3	POLD1	Exonic Synonymous SNV
CCDC6	UTR3	PREX2	Upstream
CD274	UTR3	RANBP2	Exonic Nonsynonymous SNV
CD79A	Upstream	ROBO2	Exonic Nonsynonymous SNV
CDKN1A	Downstream	RSPO3	Upstream
CNTRL	Exonic Nonsynonymous SNV	SF3B1	Upstream
CREB1	Upstream	SMAD4	Downstream
DNMT3A	UTR3	SUZ12	Exonic Nonsynonymous SNV
ELF4	UTR3	TGFBR2	Downstream
ELK4	Upstream	ZBTB16	UTR3
ELN	Exonic Nonsynonymous SNV	ZEB1	Exonic Nonsynonymous SNV
ELN	UTR3		
EPS15	UTR3		
ERCC2	UTR3		
ERCC3	Exonic Nonsynonymous SNV		

ERCC4	Exonic Synonymous SNV		
ETV5	Upstream		
EZR	Upstream		
FAM47C	Exonic Synonymous SNV		
FAT3	Exonic Nonsynonymous SNV		
FGFR2	Exonic Synonymous SNV		
FLNA	UTR5		
FLT3	Exonic Nonsynonymous SNV		
FOXP1	UTR3		
GAS7	UTR3		
GPC5	UTR3		
GPC5	UTR3		
GRM3	Exonic Nonsynonymous SNV		
H3F3A	Upstream		
HOXD11	UTR3		
IL6ST	UTR3		
JAK2	UTR3		
KAT7	Exonic Nonsynonymous SNV		
KCNJ5	Exonic Nonsynonymous SNV		
KCNJ5	Upstream		
KDM6A	Exonic Nonsynonymous SNV		
KDSR	UTR3		
KEAP1	Downstream		
KMT2A	UTR3		
KMT2C	Exonic Nonsynonymous SNV		
KMT2D	Exonic Synonymous SNV		
LZTR1	Downstream		
MAF	Upstream		
MALT1	Exonic Nonsynonymous SNV		
MALT1	Exonic Nonsynonymous SNV		
MALT1	Exonic Nonsynonymous SNV		
MAP2K4	Downstream		
MAP3K13	Exonic Nonsynonymous SNV		
MITF	Translocation Gene Fusion		
MLLT1	UTR3		
MLLT10	Exonic Nonsynonymous SNV		
MSN	Upstream		
MUTYH	Exonic Synonymous SNV		
NACA	Exonic Nonsynonymous SNV		
NACA	Exonic Synonymous SNV		
NBEA	Translocation Gene Fusion		

NF1	UTR3		
NFKB2	Exonic Nonsynonymous SNV		
NIN	Exonic Synonymous SNV		
NTRK3	Exonic Stopgain		
NUP98	Exonic Nonsynonymous SNV		
NUTM1	Exonic Nonsynonymous SNV		
PAX8	Exonic Nonsynonymous SNV		
PDGFRA	Downstream		
PDGFRA	CNV Duplication		
PDGFRB	Exonic Nonsynonymous SNV		
PHOX2B	Upstream		
PICALM	UTR3		
POU2AF1	UTR3		
PTCH1	Exonic Synonymous SNV		
PTCH1	Exonic Nonsynonymous SNV		
PTCH1	Upstream		
PTK6	Downstream		
PTPN6	Exonic Nonsynonymous SNV		
PTPN6	Exonic Stopgain		
PTPRT	Exonic Nonsynonymous SNV		
PTPRT	UTR3		
PWWP2A	Downstream		
RARA	Upstream		
REL	Upstream		
REL	UTR5		
RET	Exonic Nonsynonymous SNV		
RMI2	UTR3		
RNF213	Exonic Nonsynonymous SNV		
ROS1	Exonic Nonsynonymous SNV		
SDHAF2	UTR5		
SETD2	Exonic Nonsynonymous SNV		
SFPQ	Exonic Nonsynonymous SNV		
SIRPA	Downstream		
SIX1	UTR5		
SKI	Exonic Synonymous SNV		
SMARCE1	UTR3		
SOCS1	UTR3		
SPEN	Exonic Nonsynonymous SNV		
SRC	UTR5		
SRGAP3	UTR3		
STAG1	Exonic Nonsynonymous SNV		

STK11	Exonic Nonsynonymous SNV		
STRN	Upstream		
TAF15	Upstream		
TBX3	UTR5		
TCF3	Exonic Nonsynonymous SNV		
TEC	Upstream		
TET1	UTR3		
TET2	UTR3		
TFEB	Downstream		
TFEB	Exonic Nonsynonymous SNV		
THRAP3	Exonic Stopgain		
TMPRSS2	UTR5		
TRAF7	Exonic Nonsynonymous SNV		
TRIM24	Exonic Nonsynonymous SNV		
TRIM27	UTR5		
TRIP11	Exonic Nonsynonymous SNV		
TSC1	UTR3		
TSHR	Exonic Synonymous SNV		
VAV1	Upstream		
VHL	UTR3		
WT1	Downstream		
ZFHX3	Exonic Nonsynonymous SNV		

Supplemental Table 2: Table shows Cosmic cancer associated genes that are mutated exclusively within E2F1 KO or E2F WT mouse tumors.

Supplemental Table 3:

Gene Sets Enriched in Phenotype E2F WT

Gene Set	SIZE	ES	NES	NOM p-val	FDR q-val	FWER p-val	RANK AT MAX	LEADING EDGE
GO_MITOCHONDRION_LOCALIZATION	33	0.67	1.93	0	1	0.495	362	tags=18%, list=3%, signal=19%
GO_BETA_CATENIN_DESTRUCTION_COMPLEX_DISASSEMBLY	21	0.58	1.86	0	1	0.746	431	tags=14%, list=3%, signal=15%
REACTOME_SIGNALING_BY_FGFR1_FUSION_MUTANTS	15	0.7	1.86	0.004	1	0.717	3080	tags=60%, list=23%, signal=77%
GO_ORGANELLE_TRANSPORT_ALONG_MICROTUBULE	49	0.49	1.85	0.002	1	0.757	471	tags=8%, list=3%, signal=8%
DEN_INTERACT_WITH_LCA5	25	0.55	1.85	0.014	1	0.775	1188	tags=12%, list=9%, signal=13%
BIOCARTA_MCALPAIN_PATHWAY	21	0.72	1.83	0	1	0.811	3	tags=5%, list=0%, signal=5%
FLOTHO_PEDIATRIC_ALL_THERAPY_RESPONSE_UP	37	0.48	1.79	0.024	1	0.901	1724	tags=14%, list=13%, signal=15%
REACTOME_PI3K_EVENTS_IN_ERBB4_SIGNALING	33	0.61	1.78	0	1	0.911	2670	tags=39%, list=20%, signal=49%
BOYAULT_LIVER_CANCER_SUBCLASS_G12_UP	32	0.54	1.78	0.002	1	0.918	1	tags=3%, list=0%, signal=3%
GO_CELLULAR_RESPONSE_TO_GLUCOSE_STARVATION	26	0.59	1.78	0.018	1	0.925	2389	tags=38%, list=18%, signal=47%
GO_RIBONUCLEOPROTEIN_GRANULE	102	0.41	1.77	0.008	1	0.932	676	tags=7%, list=5%, signal=7%
KEGG_ERBB_SIGNALING_PATHWAY	75	0.5	1.76	0	1	0.954	3438	tags=39%, list=25%, signal=51%
GARGALOVIC_RESPONSE_TO_OXIDIZED_PHOSPHOLIPIDS_TURQUOISE_UP	55	0.56	1.76	0.002	1	0.951	3199	tags=38%, list=23%, signal=50%
CHIBA_RESPONSE_TO_TSA	40	0.51	1.76	0.012	1	0.954	2076	tags=23%, list=15%, signal=26%
BIOCARTA_INTEGRIN_PATHWAY	35	0.71	1.75	0.002	1	0.957	42	tags=9%, list=0%, signal=9%
GO_ENDOPLASMIC_RETICULUM_ORGANIZATION	32	0.65	1.74	0.008	1	0.966	45	tags=6%, list=0%, signal=6%
GO_RESPONSE_TO_ISCHEMIA	23	0.63	1.73	0.002	1	0.973	3360	tags=52%, list=25%, signal=69%
PID_RHOA_PATHWAY	39	0.53	1.73	0.006	1	0.975	3834	tags=41%, list=28%, signal=57%
GO_INTRINSIC_APOPTOTIC_SIGNALING_PATHWAY_IN_RESPONSE_TO_ENDOPLASMIC_RETICULUM_STRESS	31	0.62	1.73	0.01	1	0.973	1068	tags=23%, list=8%, signal=24%
PID_FAK_PATHWAY	55	0.5	1.73	0.018	1	0.971	3909	tags=40%, list=29%, signal=56%

Supplemental table 3: Table shows the top 20 enriched gene sets found in E2F WT Neu and PyMT tumors, through Gene Set Enrichment Analysis.

Supplemental Table 4:

Gene Sets Enriched in Phenotype E2F1 KO

Gene Set	SIZE	ES	NES	NOM p-val	FDR q-val	FWER p-val	RANK AT MAX	LEADING EDGE
REACTOME_RNA_POL_I_RNA_POL_III_AND_MITOCHONDRIAL_TRANSCRIPTION	52	-0.52	-2.01	0.002	0.796	0.27	3873	tags=58%, list=28%, signal=80%
GO_NUCLEOTIDE_EXCISION_REPAIR_PREINCISION_COMPLEX_STABILIZATION	19	-0.66	-1.98	0	0.577	0.365	1781	tags=42%, list=13%, signal=48%
GO_AEROBIC_RESPIRATION	42	-0.54	-1.97	0.008	0.432	0.399	5754	tags=81%, list=42%, signal=140%
GO_SINGLE_STRANDED_DNA_BINDING	70	-0.61	-1.93	0.002	0.508	0.529	1762	tags=33%, list=13%, signal=38%
GO_NUCLEOTIDE_EXCISION_REPAIR_DNA_INCISION	34	-0.67	-1.91	0	0.5	0.595	2366	tags=47%, list=17%, signal=57%
KEGG_NUCLEOTIDE_EXCISION_REPAIR	40	-0.66	-1.89	0.002	0.504	0.661	2366	tags=48%, list=17%, signal=57%
REACTOME_GLOBAL_GENOMIC_NER_GG_NER	32	-0.69	-1.88	0	0.381	0.701	2366	tags=56%, list=17%, signal=68%
REACTOME_REGULATION_OF_ORNITHINE_DECARBOXYLASE_ODC	46	-0.55	-1.88	0.013	0.424	0.699	4908	tags=72%, list=36%, signal=112%
GO_NUCLEOTIDE_EXCISION_REPAIR_PREINCISION_COMPLEX_ASSEMBLY	25	-0.6	-1.88	0.004	0.474	0.687	2176	tags=40%, list=16%, signal=48%
GO_MITOCHONDRIAL_RESPIRATORY_CHAIN_COMPLEX_ASSEMBLY	54	-0.59	-1.86	0.01	0.369	0.769	4570	tags=70%, list=33%, signal=105%
REACTOME_SIGNALING_BY_WNT	58	-0.47	-1.86	0.018	0.387	0.761	4745	tags=64%, list=35%, signal=97%
GO_PROTEIN_TARGETING_TO_MITOCHONDRION	41	-0.54	-1.86	0.019	0.414	0.756	4634	tags=71%, list=34%, signal=107%
CHNG_MULTIPLE_MYELOMA_HYPERPLOID_DN	25	-0.49	-1.85	0.013	0.297	0.803	4101	tags=48%, list=30%, signal=68%
REACTOME_FORMATION_OF_INCISION_COMPLEX_IN_GG_NER	20	-0.59	-1.85	0.01	0.309	0.8	2176	tags=40%, list=16%, signal=48%
REACTOME_NUCLEOTIDE_EXCISION_REPAIR	45	-0.62	-1.85	0.006	0.329	0.799	3410	tags=56%, list=25%, signal=74%
GO_REGULATION_OF_DNA_METHYLATION	15	-0.62	-1.85	0.006	0.35	0.799	3226	tags=60%, list=24%, signal=78%
REACTOME_TRANSCRIPTION	119	-0.44	-1.85	0.024	0.361	0.785	3913	tags=42%, list=29%, signal=58%
MA_MYELOID_DIFFERENTIATION_UP	33	-0.66	-1.84	0	0.275	0.812	367	tags=9%, list=3%, signal=9%
GO_TRANSCRIPTION_COUPLED_NUCLEOTIDE_EXCISION_REPAIR	63	-0.56	-1.84	0.006	0.285	0.807	3830	tags=54%, list=28%, signal=75%
REACTOME_SCF_BETA_TRCP_MEDIATED_DEGRADATION_OF_EMI1	45	-0.57	-1.83	0.013	0.292	0.834	4745	tags=76%, list=35%, signal=115%

Supplemental table 4: Table shows the top 20 enriched gene sets found in E2F1 KO Neu and PyMT tumors, through Gene Set Enrichment Analysis.
